# Fully automatic algorithm for the analysis of vessels in the angiographic image of the eye fundus

**DOI:** 10.1186/1475-925X-11-35

**Published:** 2012-06-22

**Authors:** Robert Koprowski, Sławomir Jan Teper, Beata Węglarz, Edward Wylęgała, Michał Krejca, Zygmunt Wróbel

**Affiliations:** 1 Department of Computer Biomedical Systems, Institute of Computer Science, University of Silesia, ul. Będzińska 39, 41-200, Sosnowiec, Poland; 2 Department of Ophthalmology, Okregowy Szpital Kolejowy, Panewnicka 65, 40-760, Katowice, Poland; 3 I Clinical Department of Cardiacurgery, Medical University of Silesia, Śląski Ośrodek Kardiologii, Katowice, Poland

**Keywords:** Image processing, Angiographic image, Fully automatic algorithm

## Abstract

**Background:**

The available scientific literature contains descriptions of manual, semi-automated and automated methods for analysing angiographic images. The presented algorithms segment vessels calculating their tortuosity or number in a given area. We describe a statistical analysis of the inclination of the vessels in the fundus as related to their distance from the center of the optic disc.

**Methods:**

The paper presents an automated method for analysing vessels which are found in angiographic images of the eye using a Matlab implemented algorithm. It performs filtration and convolution operations with suggested masks. The result is an image containing information on the location of vessels and their inclination angle in relation to the center of the optic disc. This is a new approach to the analysis of vessels whose usefulness has been confirmed in the diagnosis of hypertension.

**Results:**

The proposed algorithm analyzed and processed the images of the eye fundus using a classifier in the form of decision trees. It enabled the proper classification of healthy patients and those with hypertension. The result is a very good separation of healthy subjects from the hypertensive ones: sensitivity - 83%, specificity - 100%, accuracy - 96%. This confirms a practical usefulness of the proposed method.

**Conclusions:**

This paper presents an algorithm for the automatic analysis of morphological parameters of the fundus vessels. Such an analysis is performed during fluorescein angiography of the eye. The presented algorithm automatically calculates the global statistical features connected with both tortuosity of vessels and their total area or their number.

## Background

The available literature contains descriptions of manual, semi-automated and automated methods for analyzing angiographic images. The presented algorithms often refer to other visualization methods [1]. They segment vessels, calculate their tortuosity or number in a given area. The accompanying changes in the analyzed area of the eye fundus are also colour-coded [2], or analyzed locally (analyzed in a declared area) [3]. The local analysis of both the width of arterioles as well as the number of intersections between them is described in several papers [1,4-7]. What is usually measured is the number of intersections (decussations) of vessels, their tortuosity and diameter. It is done by, for example, IVAN software [8]. The measurement range is usually placed within the optic disc radius r - and ranges from 2r to 3r. The measurements of both tortuosity and meane diameter of arterioles in this range lead to reliable results. A good example is the Blue Mountain Eye Study which showed that the diameter of retinal arterioles and venules was decreasing with age regardless of other factors [9,10]. On the other hand, in the Beaver Dam Study, it was observed that hypertension was the cause of retinal arteriolar narrowing. However, this phenomenon turned out to be less pronounced in older patients [11]. This fact proves inadequate vascular response to hypertension in this population, which may be caused by atherosclerosis or increased vascular wall stiffness.

There exists a large number of papers connected with the analysis and processing of the eye fundus images. The methods of image analysis and processing described in them are profiled to enhance segmentation of vessels. In papers [12-16], the authors perform skeletonization of blood vessels and then present its successive approximation in accordance with the adopted algorithm. This method is extremely time-consuming because it requires the use of sophisticated techniques for the initial stage of image analysis and processing, and is also connected with a morphological operation (skeletonization) and approximation. For example, paper [12] presents a method for assessing tortuosity of vessels by their subsequent division into smaller and smaller segments. Individual vessels can also be compared with a pattern, as it was done in paper [15]. Here the authors used a pattern in the form of a sine wave with variable amplitude in order to assess vascular tortuosity. Another method for obtaining correct results of segmentation is tracking the vessel outline which is described in papers [14,17]. In paper [18], the authors describe the problems and methodology of measurements performed on images of the eye fundus and based on segmentation of vessels. In paper [19], the authors compare the results obtained from colour fundus photographs (FPs) and fluorescein angiographs (FAs). In both cases, however, the methods of image analysis refer to segmentation of vessels. Generally, the obtained results are compared with assessments of an expert whose task is to verify them - in paper [20], e.g., there are three experts. They are not always profiled algorithms for the analysis of just this type of images. Sometimes they are supported by additional software such as, for example, Paint-Shop Pro (Jasc Software) used in paper [21].

Regardless of the results, the presented methods of measuring the characteristics (diameter of the veins and arteries, the tortuosity and the number of intersections) are based on the repetitive course of the following proceedings:

· segmentation of veins and arteries as separate objects,

· calculation of average values of tortuosity or diameter of veins or arteries in a declared area.

Taking into consideration computational complexity of these two points, it can be noted that segmentation of veins and arterioles as separate objects is unnecessary as after this stage, features are combined and the average value is calculated. Bearing this in mind, the authors suggested a measurement method free of this defect [9,10,22]. When examining the number of intersections, vein and artery segmentation is not critical. However, it enables to include all vessels in the examined area in a more accurate and reliable way. In the angiographic images of the eye fundus, the vessel lumen diameter is evaluated. Whereas in the colour images, evaluation concerns the entire vessel together with the adventitia. The vessel lumen diameter better reflects the functional state of the vessel and directly influences the flow. The downside is the lack of a fully automatic distinction between arterioles and venules.

We describe a method of statistical analysis of the inclination of the vessels in the fundus related to their distance from the center of the optic disc. This method, however, differs from the classical methods published in [4,6,23]. Only the fractal analysis [24,25] is similar to the approach presented in this article. However, this similarity concerns only the global approach to the analysis.

The algorithm created by the authors should have the following characteristics:

· fully automatic image analysis - without operator intervention - even in the batch mode,

· analysis of angiographic images of the eye fundus at any resolution, both spatial resolution (pixels per inch) and pixel depth (bits per pixel),

· automatic, statistical analysis of the diameter of vessels and their degree of tortuosity,

· automatic calculation of full statistics like:

· average gradient of all vessels at any distance from the optic disc,

· average volume occupied by all vessels at any distance from the optic disc,

· automatic analysis of a group of images (for consecutive patients or for the same patient, but between successive tests): an average measure, STD, median or an average value of changes in thickness of vessels,

· auto-save to disk of the received data and images.

The proposed algorithm was tested on a group of patients described below.

## Materials

In order to verify the assumptions and correctness of the choice of the afore-mentioned image features, sample tests on patients have been performed. For this purpose, 12 healthy subjects and 40 patients with hypertension have been examined. They were aged 22 to 87 with a body weight between 53 to 92kg. Some of them were healthy and the others suffered from hypertension which was cardiologically stated. The Topcon funduscamera was used in the study. The camera is made by Zeiss and has angular width of 45 degrees and spatial resolution of 2136x3216 pixels. Fluorescein angiography was performed in a conventional way, and the images of the transit phase were analyzed as they best reflect the size of the vessel lumen. The patients agreed consciously to participate in the study which was conducted in accordance with the principles of the Declaration of Helsinki.

The algorithm suggested by the authors and the results of the analysis for the examined patients are described below.

## Method

The description of methodology for image analysis and processing is divided into a preliminary analysis of images (acquisition and filtration) and an appropriate algorithm.

### Preprocessing

L_GRAY_ angiographic image in DICOM format with a resolution MxN = 2136x3216 pixels is filtered with a median filter whose mask size is h_1_, M_h1_xN_h1_ = 3x3. The aim is to eliminate the noise. The mask size was chosen arbitrarily taking into consideration the minimum size of objects and optimization of the operation time. For the adopted size of the mask and the image resolution of 8 bits per pixel, the filtration time was 100ms. This part of the algorithm was implemented in the C language and Matlab for a PC with an Intel Xenon processor X5680@3.33GHz, 12GB RAM. L_GRAY_ image after filtration with a median filter L_MED_ undergoes successive stages of processing.

### Algorithm

**Figure 1:**
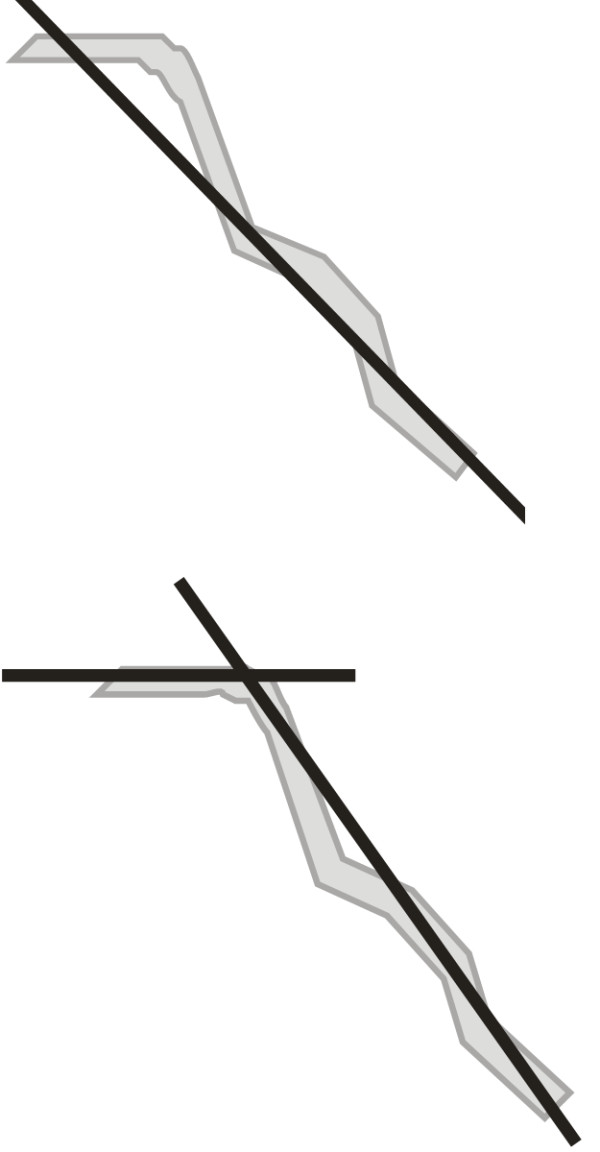
** Approximation of vessel inclination by one straight line and by two straight lines.** The approximation of a vessel by a line may be used to determine its position in space. However, in the case of a single line or of two lines this method is not accurate. Vessels should be approximated by many lines.

**Figure 2:**
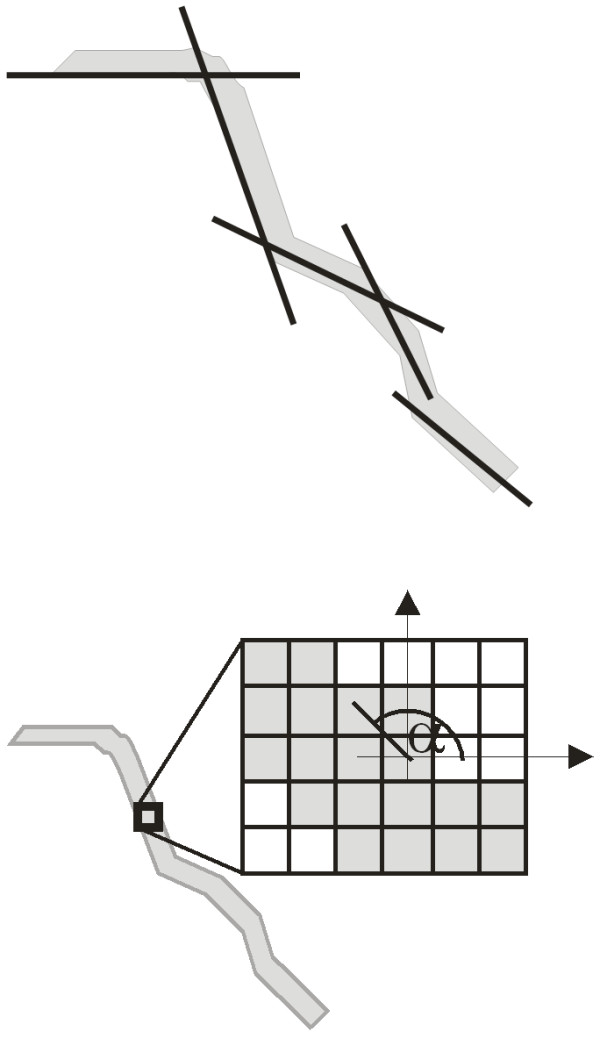
** Approximation of vessel inclination by many straight lines and approximation of vessel inclination by the value of angle of tangent inclination α at given point.** The approximation of a vessel by many lines increases the accuracy, but the computational complexity increases. In an extreme case the vessel’s position is approximated by lines 1 pixel long. So it is possible to assume that each pixel is a value of inclination angle of the tangent to the vessel at the given point.

As outlined in the introduction, it is necessary to develop an algorithm that analyzes the inclination of vessels automatically. Finding the inclination angle will enable to calculate full statistics related to the assessment of the vessel width or its tortuosity. Vessels will be analyzed in such a way that their shape will be approximated, depending on the accuracy. It will be done by one straight line (k = 1), two straight lines (k = 2), a few straight lines or by replacing each image pixel with a value of the tangent inclination angle α at a given point – as shown in Figure 1, Figure 2, respectively.

A method of this type (shown in Figure 1, Figure 2) allows for arbitrarily accurate approximation of the inclination angle (for the values k = 1,2,3 etc. up to the angle values α). By the same token, it enables to calculate the width of the vessel at a given point.

The method shown in Figure 2 shows the greatest accuracy but also the largest computational complexity. Thus, it will be still used. The set of values of the inclination angle α creates an inclination field, hereinafter referred to as L_α._ It allows for an automated analysis of vessels width which is done as accurately as possible with the level of the resolution error ±1bit.

Our proposed method of calculation of the matrix L_α_ uses convolution with a mask h_2_ created from the Gaussian function [26-28], i.e.

(1) $$h_{2}\left(m_{h2}{\text{,σ,ϵ}}_{\text{mi}}\text{,θ=}0\right)=\underset{n_{h2}}{\underset{︸}{\left[\begin{array}{c} 1\\ 1\\ \ldots \\ 1 \end{array}\right]}}*\left({\text{ϵ}}_{\text{ma}}{-\text{ϵ}}_{\text{mi}}\right)+{\text{ϵ}}_{\text{mi}}\cdot \text{exp}\left\{-\frac{m_{h2}{}^{2}}{2\cdot \sigma^{2}}\right\}$$

where:

ϵ_ma_, ϵ_mi_-- the maximum and minimum values of the mask h_2_,

θ -- the inclination angle of the mask h_2_,

m_h2_,n_h2_ -- the coordinates of individual values in the mask h_2_,

σ -- standard deviations of an average.

**Figure 3:**
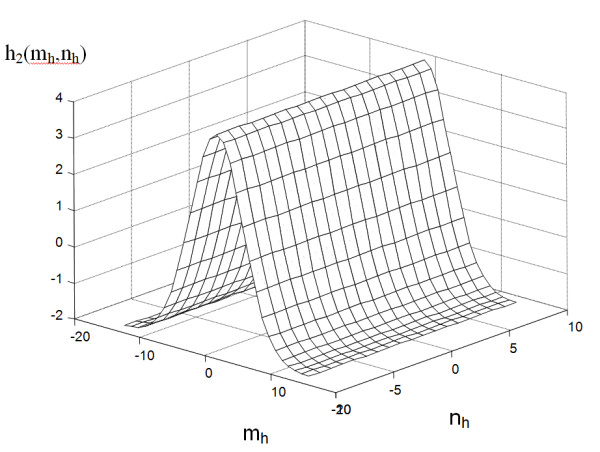
** Mask h**_**2**_**obtained for ϵ**_**mi**_ **= -2 and ϵ**_**ma**_ **= 4, σ = 1 and θ = 0°.** This mask is applied to the basic operation of convolution with the input image. The shape of this mask substantially affects the accuracy and operations speed of the algorithm. The mask size depends on the image resolution and on the vessels width range.

For example, for ϵ_mi_ = -2 and ϵ_ma_ = 4, σ = 1 and θ = 0°, and a resolution of 29x19, the values in the mask obtained are the ones shown in Figure 3.

**Figure 4:**
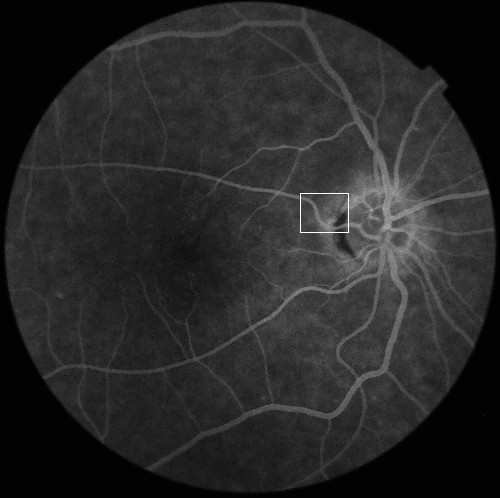
** Image L**_**MED**_**and marked fragment, on which results will be shown (Figure**5**).** Operations of image analysis and processing will be carried out in the marked area.

The next stage of the algorithm operation is to perform sequential convolution with the presented mask h_2_ in accordance with (1) for angles θ in the range 0° to 179° for every 1°. A resolution of 1° strictly determines the accuracy of the obtained results of tangent inclination angle at a given point. On the other hand, it is associated with computational complexity which is crucial for this place of the algorithm. From other results obtained for convolution L_h_, the maximum value L_ma_ and the angle for which it occurred are stored, i.e.:

(2) $$L_{h}\left(\text{m,n,θ}\right)\text{=}\sum_{m_{h2}{\text{=-M}}_{h2}/2}^{M_{h2}/2}\sum_{n_{h2}{\text{=-N}}_{h2}/2}^{M_{h2}/2}L_{\text{MED}}\left({\text{m+m}}_{h2}{\text{,n+n}}_{h2}\right){\text{·h}}_{2}\left(m_{h2}{\text{,σ,ϵ}}_{\text{ma}}{\text{,ϵ}}_{\text{mi}}\text{,θ}\right)$$

(3) $$L_{\text{ma}}\left(\text{m,n}\right)\text{=}\underset{\text{θϵ}\left(0,179\right)}{\text{max}}\left(L_{h}\left(\text{m,n,θ}\right)\right)$$

where:

m, n –the coordinate - row and column of the matrix of the input image - in this case L_MED_ (Figure 4).

**Figure 5:**
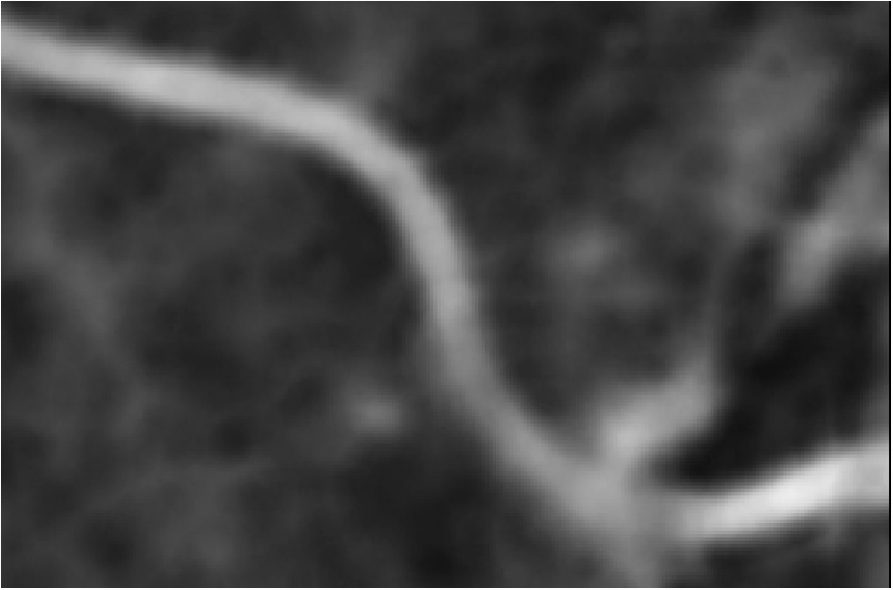
** Fragment of image L**_**ma.**_**Bright values of pixels indicate a good detection of the object.** The visible bright area will also be the basis for vessels segmentation. Together with the image from Figure 6 it will be the basis for further analyses. The degree of objects brightness will decide, whether they will be considered in further calculations.

**Figure 6:**
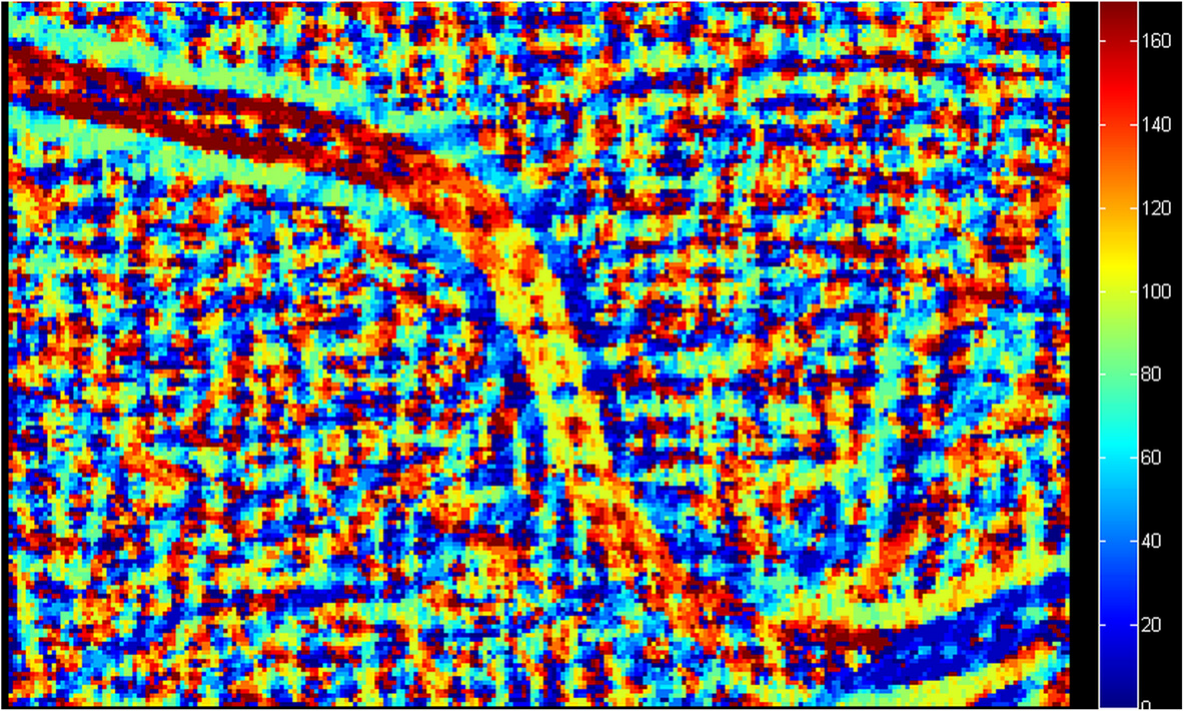
** Fragment of image L**_**θ.**_** The pixel brightness is proportional to the angular values calculated in accordance with Cartesian coordinates.** Each pixel contains information about the inclination angle of the tangent to the vessel at the given point. The presented results are the basis for further analysis. The separation of pixels to be taken into account will be carried out together with the information provided in Figure 5.

Consequently, we get two matrices L_ma_ and L_θ_ (shown in Figure 5, Figure 6). The first one contains information about the "degree of match" of the mask h_2_ to the analyzed part of the image L_MED_. The latter one shows for which mask h_2_ (ie, for which angle value θ), the match occurred. The combination of these two matrices (L_ma_ and L_θ_) enables to create an image L_w_ which contains information about the location of pixels that constitute a vessel as well as the tangent inclination angle at a given point, i.e.:

(4) $$L_{w}(\text{m,n)=}\{\begin{array}{ccc} L_{\text{θ}}(\text{m,n}) & \text{if} & L_{\text{ma}}\left(\text{m,n}\right){\text{>p}}_{r}\\ 0 & \text{if} & L_{\text{ma}}\left(\text{m,n}\right)\leq p_{r} \end{array}$$

where:

p_r_ –decimal-to-binary conversion threshold.

**Figure 7:**
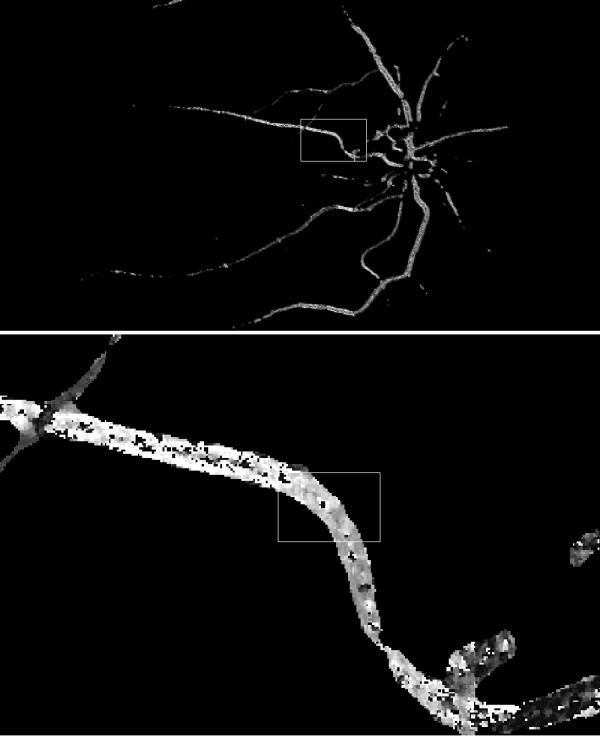
** Image L**_**w**_**and enlarged image (L**_**w**_**).** The input image resulting from the decimal-to-binary conversion of image originating from Figure 5 and multiplying it by the image from Figure 6. The black pixels are not taken into account in the computations. The other pixels indicate the value of tangent inclination angle at the given point of the vessel, showing at the same time the place of its existence.

**Figure 8:**
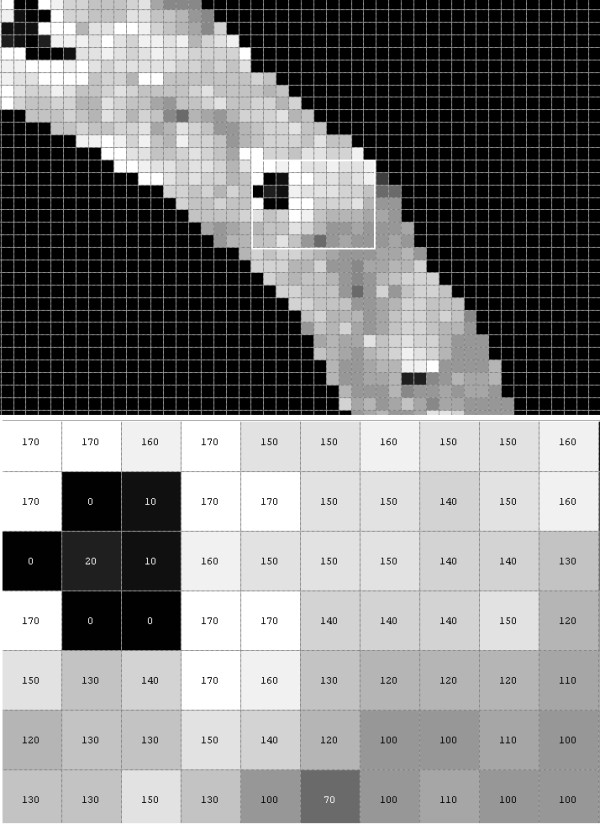
** Enlarged image from Figure**7**(L**_**w**_**) with visible values of angle θ.** Pixels of around 140 degrees value are visible on the image. They exist in the right place, where the vessel has been recognised. Their values are angular inclination values of the tangent to the vessel at the given point.

The value of the threshold p_r_ is selected automatically on the basis of the results obtained from Nobuyuki Otsu’s method [29]. The resulting image is shown in Figure 7 and its subsequent zooms in Figure 8, in which the value of the angle θ is visible.

**Figure 9:**
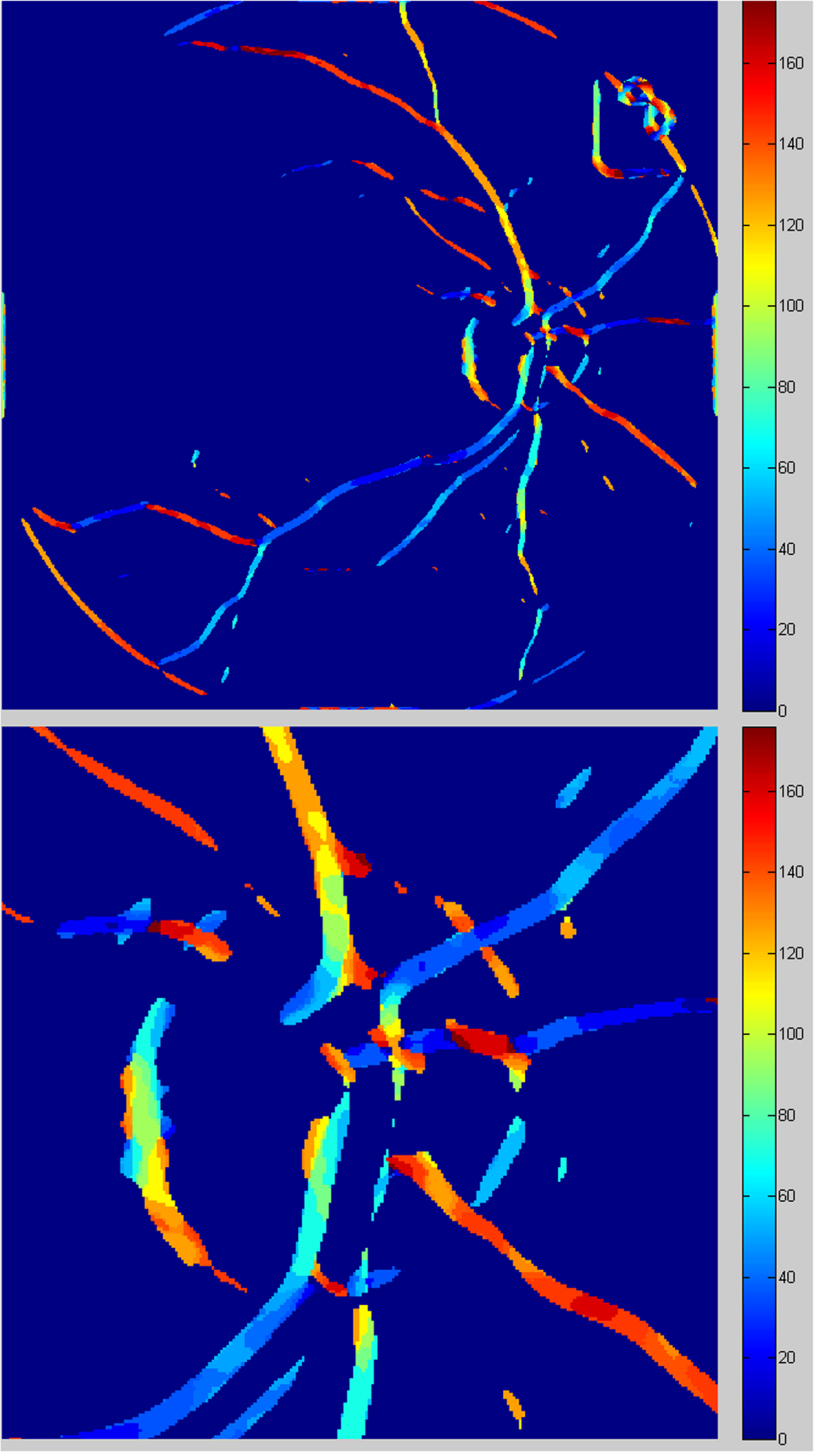
** Image L**_**w**_**with a colour palette (without the colour palette it has been presented in Figure**7**) and its enlargement.** The pixels colour is proportional to the angular values calculated in accordance with Cartesian coordinates. The objects featuring a blue colour have an inclination between 0° and approx. 50°, 60°, green and yellow colours mean a nearly vertical arrangement and the red stands for objects, which inclination substantially exceeds 140.

**Figure 10:**
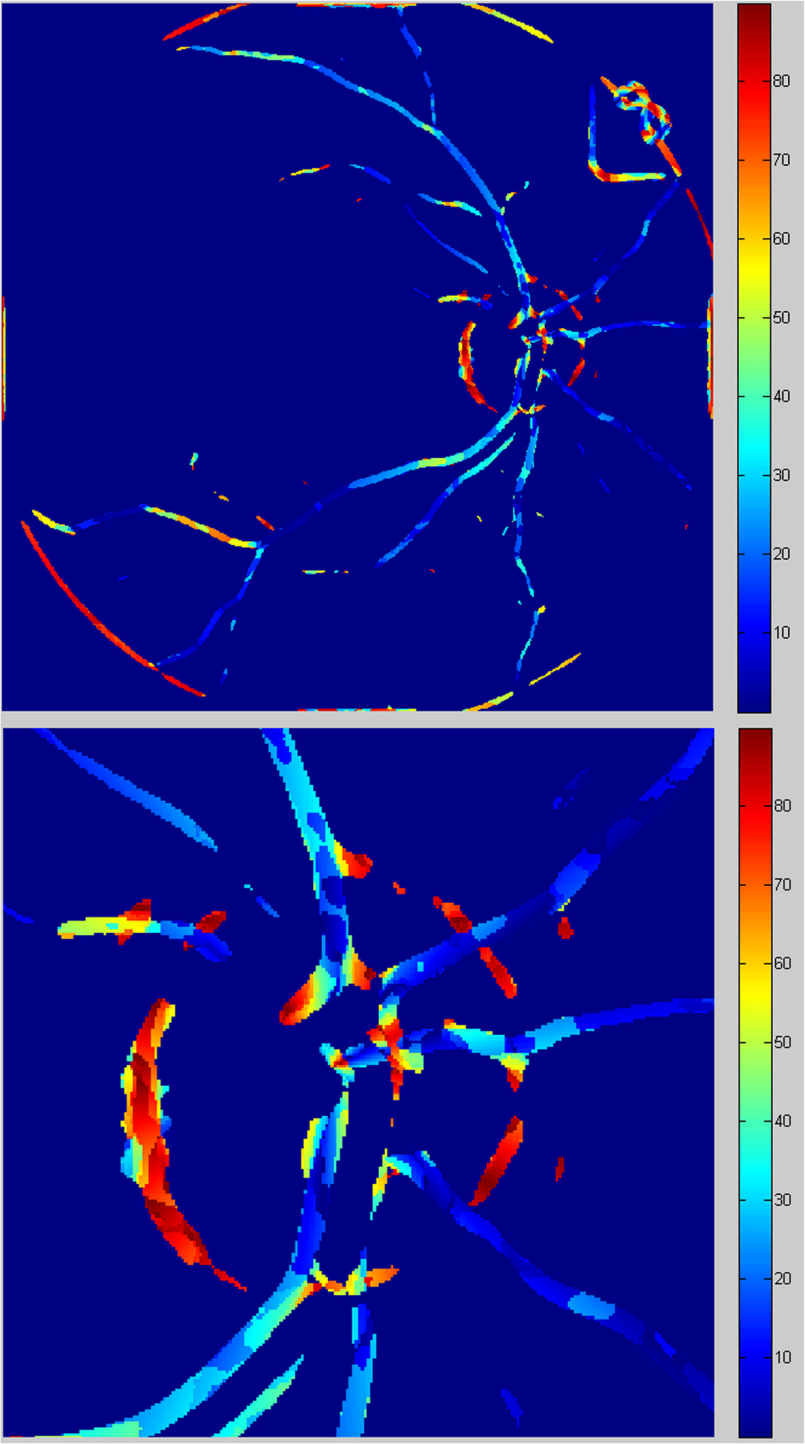
** Image L**_**k**_** and its enlargement.** The pixels colour is proportional to the angular values calculated in accordance with Cartesian coordinates, which centre is located in the centre of the optic disc. Additionally, the angular values are modified and indicate the angle measured against the radius. The blue objects radiate from the centre of the optic disc; the red objects are arranged perpendicularly to the radius going out from the centre of the optic disc.

The pixel values in the image L_w_ should be corrected accordingly. Owing to this correction, it is possible to modify the angular values in such a way that they indicate the inclination angle with respect to a circular coordinate system, whose center is located in the center of the optic disc. The angular values (in the range of 0 to 90°) obtained in this way are reliable in relation to the assessment of tortuosity of segmented vessels. A part of the image L_w,_ before and after correction L_k,_ is shown in Figure 9 and Figure 10.

The image L_k_ represents the final form of the image of the algorithm for image analysis and processing. One of the possible applications of the presented algorithm, that is the measurements made in the image L_k_, is presented in next section.

## Results

The algorithm presented in the previous chapter is profiled for automatic analysis of the width of vessels. The analysis is made for each pixel of the image, with an accuracy described by the relation (1) (for the angular values with a resolution of one degree). The use of this automated method of analysis of data obtained in the image L_k_ will be suggested below.

**Figure 11:**
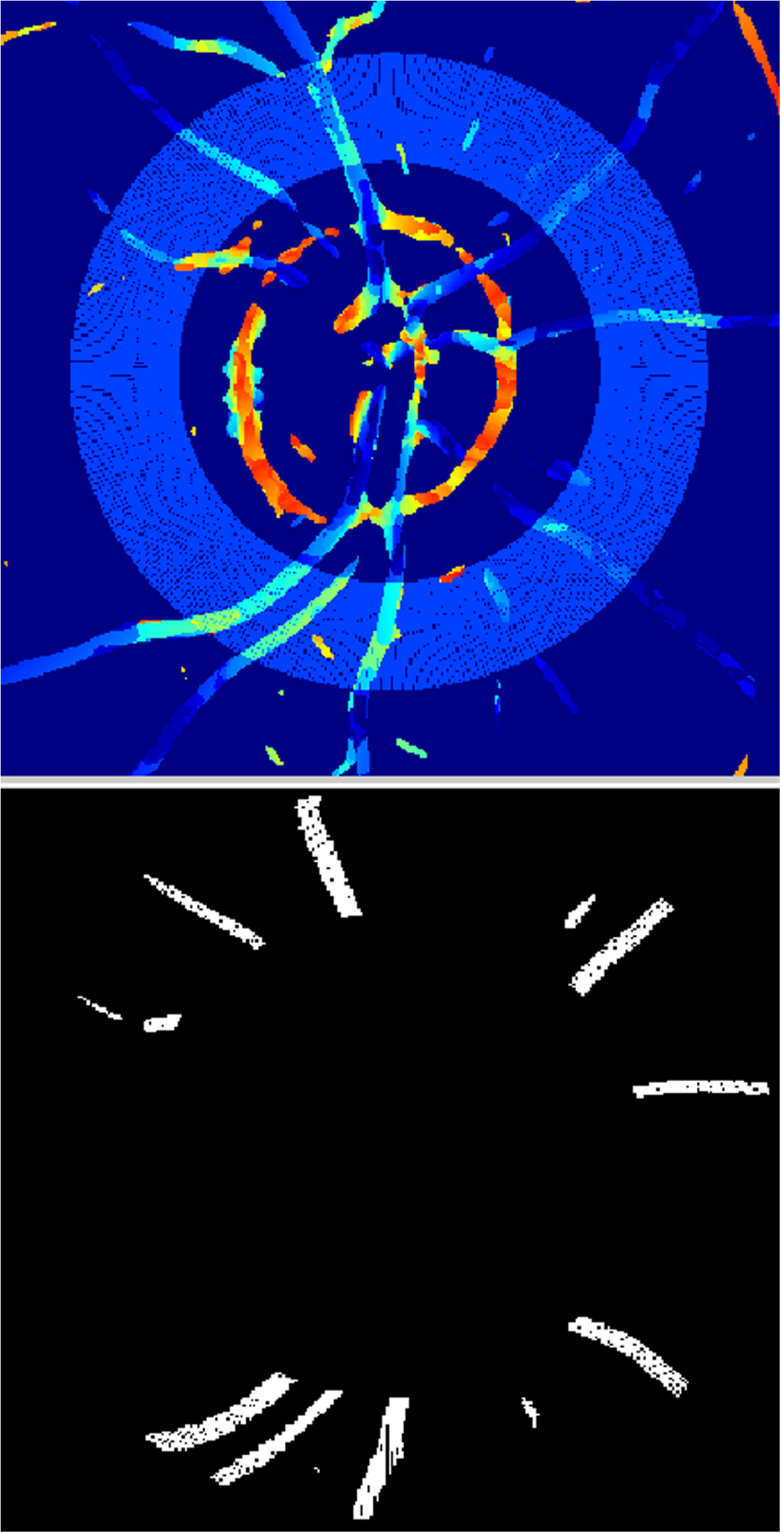
** Enlarged image L**_**k**_**with marked analysis area from 2r to 3r of optic disc radius and automatically selected fragment of analysis comprising the range from 2r to 3r of optic disc radius. Only objects (vessels) visible in the analysed area (2r to 3r) will be further analysed.** The further analysis will be related to the determination of a histogram on their basis. The black pixels visible on the image in the marked area (2r to 3r) will not be considered.

Assuming that the radius of the optic disc is known (referred to as r), we can designate a circular band whose diameter is in the range of 2r to 3r (Figure 11). This range has been proposed in IVAN software [8] and is commonly used in the calculation of changes in diameter of arteries and veins, which enables to assess the coexistence of those changes with the progression of vascular disease.

The presented analysis applies to:

fully automatic measurement without any operator intervention,

measurement of the number of vessels in a declared area further on denoted as z,

the average angular value for individual objects in the image L_k_ – further on denoted as φ_sr_,

standard deviation of mean angular values in the image L_k_ - further on denoted as φ_STD_,

calculation of the maximum value of the angle in the image L_k_ - further on denoted as φ_max_,

calculation of the percentage of the ratio of vessels surface area in relation to the measured area (calculated as the ratio of the total number of pixels that make up the vessels in the measured area to the total number of pixels of the area) – denoted as p_s,_

designation of histogram of pixel inclination of objects.

The results obtained for the test group (for healthy people and those with arterial hypertension) are given in Table 1.

**Table 1:** Fragment of results of automated measurement of morphometric parameters of vessels

**No**	**hypertension**	**z**	**Φ**_**sr**_**[**^**o**^**]**	**Φ**_**STD**_**[**^**o**^**]**	**Φ**_**max**_**[**^**o**^**]**	**p**_**s**_**[%]**
1	No	14	20.4	12.7	8	8.9
2	No	14	16.7	12.1	7	7.0
3	No	16	22.1	16.7	11	9.1
4	No	11	16.1	8.4	13	6.6
5	No	13	18.5	14.2	3	7.8
6	Yes	35	31.1	23.7	25	20.7
7	Yes	38	43.0	25.5	21	35.6
8	Yes	39	40.2	25.2	27	38.9
9	Yes	38	32.2	24.2	1	17.5
10	No	24	30.2	26.6	3	19.9
11	No	4	19.0	20.9	1	3.3
12	No	8	41.4	26.0	16	5.2
13	No	14	32.4	25.7	1	9.3
14	No	17	30.8	25.5	5	9.1

Analyzing these results, we can, for example, read them for measurement no. 4. This is a patient without hypertension for whom 11 separated objects have been detected automatically in the area 2r to 3r. Their average angle of inclination with respect to the axis was 16.1°. In addition, most pixels of objects were found for a 13° angle. All the detected pixels of objects constituted 6.6% of the total measured area (2r to 3r).

**Figure 12:**
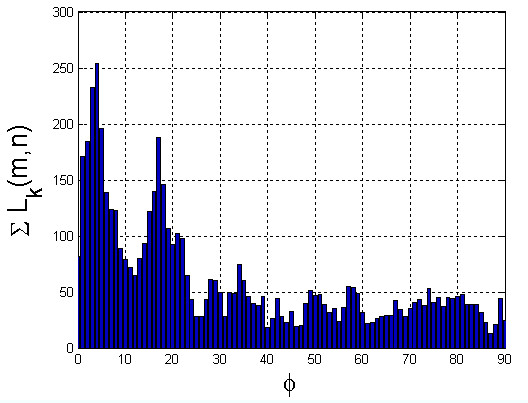
** Histogram of marked analysis area from 2r to 3r.** From the histogram ϕ_max_ = 6° for 240 pixels can be easily read. Such histogram is determined for each analysed patient, for which the most important element is the inclination angle ϕ value, which occurred for the highest bar.

In terms of diagnosis of hypertension, there is another interesting histogram which relates to changes in the values of the angle ϕ. The histogram presented in Figure  12 shows that the maximum is for the angle ϕ=6°, for which there were 240 pixels in total making up the vessels. Sloping nature of the envelope of the histogram is evidence of a small number of pixels with the value of the angle ϕ much exceeding 40°. It means that the vessels radiate from the center of the optic disc. Any angular error of unevenness is in the range of 10 to 40° and affects less than half of the pixels.

A group of 52 patients (including 40 healthy and 12 hypertensive ones) was divided in equal proportions into learning, validation and test group). Then, the cross-validation method was used, with particular reference to the ratio between the decision classes (stratified cross-validation). A classifier in the form of a decision tree was implemented, assuming five attributes: the angle for the maximum number of pixels - ϕ_max_, standard deviation of the angle average ϕ_STD_, the average value of the angle - ϕ_sr_, the number of vessels in a declared area – z, and the percentage of the ratio of vessels surface area to the measured area - p_s_. It was recognized that these attributes are equally privileged. On this basis, six decision trees were constructed; five trees for each of the attributes occurring independently and one for all of them together.

In all cases, a non-parametrical algorithm CART (Classification and Regression Trees) creating binary trees is used as the method for their induction. An increase in the nodes purity has been used as the criterion for assessing the quality of CART divisions. The Gini index has been used as the measure of nodes impurity. Because of a small number of cases, the tree creation was not limited by a minimum number of vectors in a node. Then, to prevent excessive fitting to the data, the created tree is pruned to the maximum extent. At the first stage, the resubstitution error for various subsets of the original tree has been calculated. Then the cross-validation error for these sub-trees has been calculated. The cut-off value was set at the minimum cost (misclassification error) plus one standard error. The best level has been determined as the smallest tree below this cut-off. After pruning trees (to avoid over-fitting), the following results were obtained (true positive-TP, true negative - TN, false negative – FN and false positive-FP), i.e.:

(5) $$\begin{array}{lllll} - & z & \text{TP}=12,\; & \text{TN}=37,\; & \text{FN}=0\;\text{and FP}=3,\\ - & \phi \text{SR} & \text{TP}=12, & \text{TN}=36, & \text{FN}=0\;\text{and FP}=4,\\ - & \phi \text{STD} & \text{TP}=9, & \text{TN}=40, & \text{FN}=3\;\text{and FP}=0,\\ - & \phi_{\max} & \text{TP}=10, & \text{TN}=40, & \text{FN}=2\;\text{and FP}=0,\\ - & p_{s} & \text{TP}=12, & \text{TN}=37, & \text{FN}=0\;\text{and FP}=3,\\ - & z,\;\phi \text{SR},\;\phi \text{STD},\;\phi_{\max},\;p_{s} & \text{TP}=10, & \text{TN}=40, & \text{FN}=2\;\text{and FP}=0. \end{array}$$

**Figure 13:**
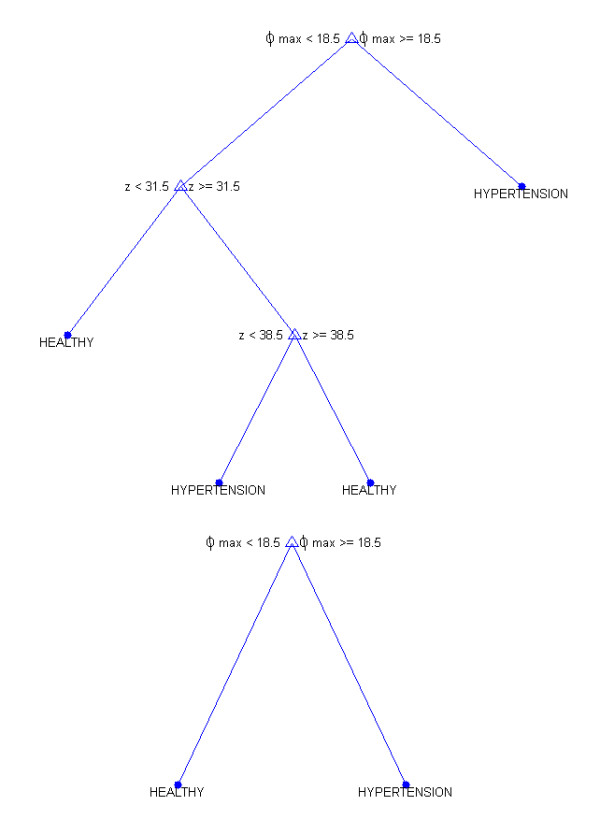
** A full decision tree and after pruning - constructed based on 5 attributes - z, Φ**_**SR**_**, Φ**_**STD**_**, Φ**_**max**_**, p**_**s**_. For the pruning decision tree only one representative attribute is obtained - ϕ_max_ - and the following classification results: TP = 10, TN = 40, FN = 2 and FP = 0.

For each of six trees created, the resultant values of accuracy have been calculated, ACC = (TP + TN)/(TP + TN + FP + FN), i.e.: z- 0.94, ϕ_SR_- 0.92, ϕ_STD_- 0.94, ϕ_max_- 0.96, p_s_- 0.94 and z, ϕ_SR_, ϕ_STD_, ϕ_max_, p_s_- 0.96. The ACC value was minimal for two pruned trees created on the basis of feature ϕ_max_ only and of all z, ϕ_SR_, ϕ_STD_, ϕ_max_ and p_s._ The tree, whose construction requires only one feature ϕ_max_, was chosen from these two decision trees. Therefore, this tree was classified as the best. In addition, after pruning, the tree constructed for z, ϕ_SR_, ϕ_STD_, ϕ_max_, p_s_ has only one node with the same attribute ϕ_max_ – which confirms its proper selection (Figure 13). For this tree (created on the basis of the attribute ϕ_max_), the following indicators are obtained: sensitivity or true positive rate TPR = TP/(TP + FN) = 0.83, false positive rate FPR = FP/(FP + TN) = 0, accuracy ACC = (TP + TN)/(TP + TN + FP + FN) = 0.96, specificity SPC = TN/(FP + TN) = 1, positive predictive value PPV = TP/(TP + FP) = 1, negative predictive value NPV = TN/(TN + FN) =0.95, false discovery rate FDR = FP/(FP + TP) = 0. Therefore, when analyzing the results obtained from the created decision tree, the average value of ϕ_max_ is 6.8±5.1° for patients without hypertension and 24.3±3° for patients with hypertension. While treating the two cases, FN = 2, as thick errors, the result obtained for all patients with hypertension is 21.6±7.6°. Calculations were made for the cut off marked from a decision tree whose ϕ_max_ = 18.5°. With a confidence level of 0.001, the critical value of Student-t distribution for a group of healthy subjects (39 degrees of freedom) is 3.55, for patients with hypertension (11 degrees of freedom) - 4.43. For the latter group, the bottom end of the confidence interval is 21.6 - 4.43 * 7.6/√ 12 = 14.5, whereas the top one is 6.8 - 3.55 * 5.1/√ 40 = 9.66. Both bands have no elements in common - they are well separated. Thus, it can be said, with probability equal to 99.9%, that the value of the angle ϕ_max_ is reliable in the assessment of hypertension. However, there are two things which should be borne in mind, namely a relatively small number of subjects with hypertension and a number of cases of false positives equal to 2, which constitutes (FN/TP * 100) 16.6% error with respect to all patients.

### Comparison with other methods

The presented algorithm is designed to collect statistical information about the number and tortuosity of vessels in the analyzed area of the eye. The value of the angle ϕ obtained from the image is, by definition, the measure of tortuosity and number of vessels. According to the information given in the introduction, the aim of the algorithm is not the most accurate segmentation of vessels. In general, objects (vessels) visible in the picture L_w_ or L_k_ (Figure  9, Figure 10) do not have to be continuous. Furthermore, the algorithm does not have to segment all the vessels. Therefore, high accuracy is not required. The only common feature is related to the fact that the ratio of thickness of vessels must be preserved. The reason is that each pixel of an object (vessel) affects the shape of the histogram (Figure 12) and thus the value ϕ_max_.

The results of the presented algorithm were compared with the results obtained with DRIVE database [30]. The assessment of quality of segmentation of vessels should be distinguished from the evaluation of the adopted statistical methods. Minor differences were obtained in the detected level of details of vessels in favor of the method described in paper [30]. Underestimation and lack of vessels continuity were the main reasons for these differences. For 20 verified cases, the underestimation was less than 15% of the total area of all objects. It should be noted that despite receiving seemingly worse results of segmentation of vessels, the method described above has not been profiled for this purpose. This method enables to obtain directly the measurement results of tortuosity and percentage of vessels in the analyzed area. Therefore, in contrast to the methods described in the introduction [12–21], there is no need to perform additional analyzes like zooming vessels with a curve [12,16] or using patterns of tortuosity [15] etc. This is the biggest advantage of the presented algorithm over other methods described in all the publications [12–21]. An additional advantage of this algorithm is negligible sensitivity to change of image acquisition parameters - for different operators, different camera settings and different patient settings. This is due to the characteristics of the algorithm: automatically corrected unevenness of lighting and acceptance of lack of vessels continuity.

Comparing the obtained results with those of other authors, a similar global approach in the fractal analysis can be found. The fractal dimension shown in papers [31,32] allows for a group division into healthy subjects and those with hypertension. In paper [31], the value, i.e. fractal dimension, is fixed at 1.437 with a standard deviation of 0.025. However, this method is semi-automatic. Paper [33], on the other hand, presents an interesting method based on nonlinear orthogonal projection approach. This method uses the afore-mentioned DRIVE database. The authors have obtained 96.1% accuracy. The results are similar to those obtained in this paper (96% accuracy). However, they were obtained with a slightly different method. The differences consist in the fact that the algorithm presented in paper [31] is not fully automatic (the differences are thus related to the segmentation method). The algorithm presented in paper [33] does not apply directly to the detection of patients with hypertension. The level of accuracy at 96.1% indicated by the authors concerns the quality of segmentation of vessels. It is not a measure of the quality of separation of patients with hypertension from healthy subjects. Yet in paper [32] a global fractal analysis applies only to chronic kidney disease. The results in the diagnosis of this condition are at 95%. Therefore, a comparison with the use of the fractal dimension was carried out for the images obtained in this study. For this purpose, Fracllac software (Local Connected Fractal Dimension Analysis function) was used, which is, for example, described in paper [24]. However, using only Fracllac software, no correlation between hypertension and the fractal dimension in the angiographic image was obtained. The reason was a major influence of lighting unevenness and artifacts visible in the image, which were not filtered. Whereas using the image pre-processing suggested in this paper, the accuracy was 81%. However, this result was obtained for the hybrid method which combines a filtration method suggested in this paper with the fractal analysis made with Fracllac software.

The algorithm described here can also be divided into functions related to each analysis phase. Then it will be possible to make a comparison with other GUI profiled to Matlab. Such an example is Fraclab [25] which is a set of functions extending the functionality of Matlab. It has common features with the presented algorithm only in terms of filtration. The main way of calculating the matrices L_ma_ and L_θ_, and on their basis L_w_, which is presented above (4), is not available there. Of course the angle of the mask h_2_ can be changed manually, and then this manual method is similar to the one presented above.

## Conclusions

This paper presents a tool (algorithm) designed for automatic analysis of morphological parameters of vessels in the fundus watched during fluorescein angiography. The presented algorithm automatically calculates the global statistical features connected with both tortuosity of vessels as well as their total area or their number. On the basis of preliminary studies, we have shown correlations between the total value of the inclination angle of vessels and hypertension. This result confirms the usefulness of the described algorithm for image analysis and processing in medical practice. However, further research in a larger population is needed. The algorithm can also operate in a batch mode where the operator only selects a folder with images for analysis. Currently, with no time optimization, the analysis of one image takes a few seconds on the Intel Core 2 Quad Q9300 2.5 GHz CPU with 8 GB RAM.

## Competing interests

The authors declare that they have no competing interests.

## Authors’ contributions

RK and ZW suggested the algorithm for images analysing and processing, implemented it and analysed the images. SJT, BW, EW performed the acquisition of the fundus of the eye images and consulted the obtained results. MK expressed opinions on the obtained results from a cardiologic point of view. All authors have read and approved the final manuscript.
